# Inherited genetic effects on arsenic metabolism: A comparison of effects on arsenic species measured in urine and in blood

**DOI:** 10.1097/EE9.0000000000000230

**Published:** 2022-11-11

**Authors:** Lizeth I Tamayo, Yohhan Kumarasinghe, Lin Tong, Olgica Balac, Habibul Ahsan, Mary Gamble, Brandon L Pierce

**Affiliations:** a Department of Public Health Sciences, University of Chicago, Chicago, IL; b Department of Statistics, University of Chicago, Chicago, IL; c Department of Environmental Health Sciences, Mailman School of Public Health, Columbia University, New York, NY; d Department of Human Genetics, University of Chicago, Chicago, IL; e Comprehensive Cancer Center, University of Chicago, Chicago, IL; f Department of Medicine, University of Chicago, Chicago, IL.

## Abstract

Inorganic arsenic (iAs) is a carcinogen, and chronic exposure is associated with adverse health outcomes, including cancer and cardiovascular disease. Consumed iAs can undergo two methylation reactions catalyzed by arsenic methyltransferase (*AS3MT*), producing monomethylated and dimethylated forms of arsenic (MMA and DMA). Methylation of iAs helps facilitate excretion of arsenic in urine, with DMA composing the majority of arsenic species excreted. Past studies have identified genetic variation in the *AS3MT* (10q24.32) and *FTCD* (21q22.3) regions associated with arsenic metabolism efficiency (AME), measured as the proportion of each species present in urine (iAs%, MMA%, and DMA%), but their association with arsenic species present in blood has not been examined. We use data from three studies nested within the Health Effects and Longitudinal Study (HEALS)—the Nutritional Influences on Arsenic Toxicity Study, the Folate and Oxidative Stress study, and the Folic Acid and Creatine Trial—to examine the association of previously identified genetic variants with arsenic species in both urine and blood of 334 individuals. We confirm that the genetic variants in *AS3MT* and *FTCD* known to effect arsenic species composition in urine (an excreted byproduct of metabolism) have similar effects on arsenic species in blood (a tissue type that directly interacts with many organs, including those prone to arsenic toxicity). This consistency we observe provides further support for the hypothesis the AME SNPs identified to date impact the efficiency of arsenic metabolism and elimination, thereby influencing internal dose of arsenic and the dose delivered to toxicity-prone organs and tissues.

**What this study adds**

The association of inherited genetic variation in the *AS3MT* and *FTCD* regions with arsenic metabolism efficiency (AME, measured in urine) is well established. However, there has been limited research examining genetic associations with arsenic species measured in blood. In this work, we show that inherited genetic variants known to impact the composition of arsenic species in urine (an excreted byproduct) have similar effects on arsenic species measured in blood (a toxicity-relevant tissue). This consistency provides additional support for the hypothesis AME-associated SNPs impact toxicity risk through their effects on internal dose of arsenic.

Inorganic arsenic (iAs) is a known human carcinogen, and chronic exposure to iAs is associated with adverse health outcomes, including diabetes, cardiovascular disease, various types of cancer, and overall mortality.^1^ Globally, chronic arsenic exposure through consumption of contaminated drinking water is a major public health issue, with an estimated 130 million people affected worldwide, including ~50 million in Bangladesh.^2^

Arsenic consumed through drinking water generally occurs as inorganic arsenic (iAs) and is primarily metabolized in the liver and in other organs to a lesser extent. iAs in its pentavalent state (As^V^) can be reduced to its trivalent state (As^III^),^3,4^ which can then undergo oxidative methylation by arsenic methyltransferase (*AS3MT*), producing monomethylarsonic acid (MMA^V^), which can in turn be reduced to monomethylarsonous acid (MMA^III^). A second methylation reaction produces dimethylarsinic acid (DMA^V^).^5^ DMA has a shorter circulating half-life and is more readily excreted in urine than MMA and iAs,^6,7^ with DMA making up the majority of excreted arsenic species.^8,9^ Therefore, an individual’s arsenic metabolism efficiency (AME) can be defined as their ability to methylate arsenic and produce DMA. The proportion of arsenic species in urine that are DMA (i.e., DMA%) is often used as a measure of AME.^10–12^

Although drinking water is one potential source of arsenic exposure, past studies have also shown that food can contribute to exposure to both inorganic arsenic and organic arsenic (i.e., rice^13,14^ and seafood^14,15,16^), with metabolism of organic forms potentially contributing to DMA. Significant levels of airborne arsenic exist in areas with industrial activity and therefore may also serve as a source of exposure.^17^

Previous studies have characterized inter-individual variation in urine-based measures of AME (e.g., DMA%).^10^ Some variation in AME can be explained by genetic factors.^11^ Prior GWA studies of Bangladeshi individuals have identified independent associations of four SNPs (single nucleotide polymorphisms) with AME: three in the 10q24.32/*AS3MT* region (rs4919690, rs11191492, and rs191177668) and one in the *FTCD* gene (rs61735836).^12,18,19^ The *FTCD* gene plays an important role in histidine catabolism, which generates one-carbon units that can enter the one-carbon/folate cycle, which in turn provides methyl groups for arsenic metabolism.^12^ All four of these SNPs are independently associated with urine DMA%.

All prior studies of the effects of inherited genetic variation on AME have exclusively used urine as a biospecimen source to measure arsenic species. There is a large difference in the distribution of As metabolites in blood compared with urine, as blood tends to have higher MMA% and much lower DMA% compared with urine due to the shorter circulating half-life of DMA.^20^ Genetic effects on arsenic species composition in other types of human biospecimen have not been assessed, so it is unknown if SNPs associated with urine-based measures of AME show similar associations with analogous measures in other tissue types. In particular, due to the large difference in the distribution of As metabolites in blood compared with urine (with relatively more MMA in blood and relatively more DMA in urine),^20^ heterogeneity in the genetic effects on the concentrations of each metabolite across blood and urine remains plausible.

In this study, we use blood as an alternative biospecimen source to assess the effects of previously identified AME SNPs on arsenic species composition. In particular, we evaluated the consistency between SNP associations with DMA% measured in urine and SNP associations with DMA% measured in blood. We also examined correlation among arsenic species measurements in blood and urine and their associations with participant characteristics.

## Methods

### Participants

The data collected for this study was obtained from a subset of individuals who participated in the Health Effects and Longitudinal Study (HEALS), a prospective epidemiologic investigation that sought to determine the effects of arsenic exposure on multiple health outcomes.^21^ HEALS examined a cohort of 11,746 adults in Araihazar, Bangladesh, a rural area where residents were exposed to high levels of arsenic through naturally contaminated drinking water.^22,23^ Some HEALS participants also participated in additional studies which investigated correlates of AME and folate interventions to increase AME. For this study, we use data from the 334 genotyped HEALS participants who additionally participated in one (or more) of three such studies: Nutritional Influences on Arsenic Toxicity (NIAT),^24^ the Folic Acid and Creatine Trial (FACT),^25^ or Folate and Oxidative Stress (FOX).^7^ These studies all measured participants’ arsenic and arsenic metabolite concentrations in both blood and urine. Data on blood arsenic species were available for 284 of the 334 genotyped individuals in the NIAT, FOX, and FACT studies. Biospecimens were collected at baseline (Week 0) for NIAT, Fox, and FACT studies and again twelve weeks after the initiation of the interventions (Week 12) for NIAT and FACT participants.

### Arsenic measurements

Concentrations of urinary arsenic species were measured using high-performance liquid chromatography (HPLC) and detected by inductively coupling plasma mass spectrometry with dynamic reaction cell technology (ICP-MS-DRC).^20^ A similar method was used to measure concentrations of blood arsenic metabolites. The LOD for arsenic species was 0.2 μg/L for iAs_III_, iAs_V_, MMA, and DMA.^18,21,26–29^ Total urine and blood arsenic was then estimated by computing the sum of arsenic metabolites iAs_III_, iAs_V_, MMA, and DMA. Urinary creatinine concentration was measured using a colorimetric technique based on the Jaffe reaction.^30^ A creatinine-adjusted total arsenic concentration, measured in μg/g creatinine, was obtained by dividing total urinary arsenic concentration by urinary creatinine concentration.

### Arsenic metabolism variables

For both urine and blood arsenic species measurements, we created variables representing AME by dividing the concentration of each major species (iAs, MMA, and DMA) by the sum of those species, to obtain the amount of each species present (iAs%, MMA%, and DMA%) as a proportion of total arsenic species. Detected arsenosugars were not included in these calculations. *A*rsenic species percentages in urine (i.e., DMA%, MMA%, iAs %) did not involve adjustment for creatinine.

### Genotyping and quality control

A subset of HEALS participants (n > 5,000) have been genotyped previously using Illumina arrays (either the HumanCyto12 300K array, or the Infinium multiethnic EUR/EAS/SAS 1.4M array). For both arrays, we measured SNP and sample quality by removing non-rs SNPs, SNPs with a call rate of <90%, monomorphic SNPs, and samples with a call rate <90%. The Michigan Imputation Server was used to conduct genotype imputation using the Haplotype Reference Consortium (HRC).^31^ Only high-quality imputed biallelic SNPs (imputation *r*^2^ > 0.3) and SNPs with minor allele frequency >0.005 were retained. From this dataset, we extracted data for our four SNPs of interest.

### Statistical methods

Pearson’s correlation and linear regressions were used (in R) to assess the relationships among blood and urine concentrations of total arsenic and arsenic species. Multivariate linear regression models were used to assess associations between measures of arsenic species and participant characteristics. Multivariate models included sex, age, BMI, smoking status, and categorical variables for each ancillary study. To analyze arsenic measurements taken across multiple timepoints, we used mixed-effects models with the proportion of each arsenic species from both week 0 and week 12 as the outcome, treating individual as a random effect. This approach enabled us to use repeated measures to increase the power of our genetic analyses.

Multivariate linear regression analyses were also used to assess independent associations between our SNPs of interest and the concentration of each metabolite in blood and urine. All four SNPs were included in single regression model, allowing us to estimate the independent association between each SNP and relative metabolite abundance. Age and sex were also included as covariates. A similar model was used to estimate SNP associations with total arsenic concentration in both blood and urine and to assess the interaction of sex with each SNPs of interest.

## Results

### Participant characteristics

Measurements of arsenic species in blood and urine were obtained for 334 genotyped HEALS participants who also participated in one of three additional studies: FACT (n = 125), FOX (n = 78), or NIAT (n = 131). Males (55%) and individuals who never smoked (60%) made up more than half of the participants (Table 1). The mean age and mean BMI at baseline were 40.8 years and 20.1 kb/m^2^ respectively.

**Table 1.** Characteristics of the 334 genotyped HEALS participants with arsenic species measured in blood and urine as a part of the FACT, FOX, or NIAT studies

Participant characteristics	n (%) or mean (SD/IQR)
**Study**	
FACT	125 (37.4%)
FOX	78 (23.4%)
NIAT	131 (39.2%)
**Sex**	
Male	184 (55.1%)
Female	150 (44.9%)
**Smoking status**	
Current or past	133 (39.8%)
Never	201 (60.2%)
**Age in years**	
Mean (SD)	40.8 (9.8)
Median (IQR)	40 (33, 48)
Range (min, max)	45 (21, 66)
**BMI (kg/m**^**2**^)	
Mean (SD)	20.1 (3.0)
Median (IQR)	19.6 (18.0, 21.7)
Range (min, max)	18.3 (14.2, 32.5)

BMI indicates body mass index; FACT, Folic Acid and Creatine Trial; FOX, folate and oxidative stress; NIAT, nutritional influences on arsenic toxicity.

### Arsenic species distributions

**Figure 1.**
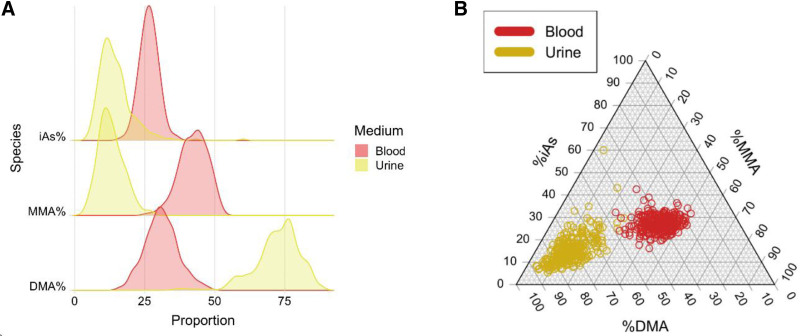
Distribution of arsenic species percentages in blood (red) and urine (yellow/gold) at baseline, shown as a ridge plot (A) and a triangle plot (B).

Each arsenic species percentage showed an approximately normal distribution in blood and urine (Figure 1A and Supplementary Figure 1A; http://links.lww.com/EE/A205). On average, DMA comprised a much greater proportion of total arsenic species in urine (72.4%) than in blood (31.1%). In contrast, iAs and MMA each accounted for a greater proportion of total arsenic species in blood (26.5% and 42.4%, respectively) compared with urine (14.6% and 13.0%). Additionally, urine appeared to have outliers which were not as apparent in blood (Figure 1B and Supplementary Figure 1B; http://links.lww.com/EE/A205).

### Associations among arsenic measurements

**Figure 2.**
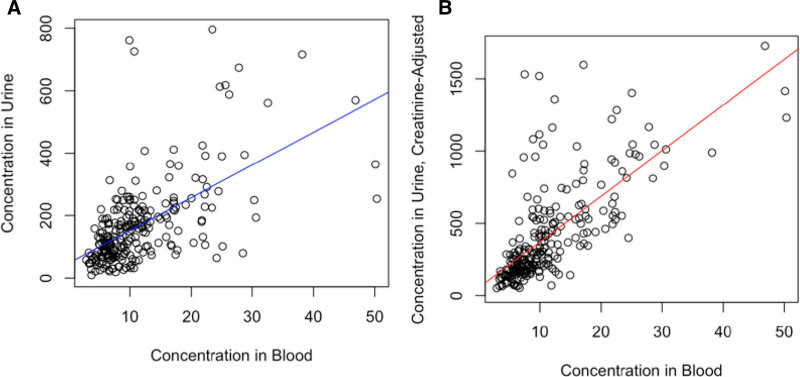
Association between urinary total arsenic and blood total arsenic without creatinine adjustment (A) and with creatinine adjustment (B) at baseline. Blood and urine total arsenic were estimated by taking the sum of iAs, MMA, and DMA.

Total arsenic concentration in blood was strongly associated with total arsenic concentration in urine (*P* < 2 × 10^–16^), and a moderate positive correlation (r = 0.58) was observed (Figure 2A). After adjusting urine arsenic concentration for creatinine, the strength of this association increased (*P* < 2 × 10^–16^), showing a stronger positive correlation (r = 0.70) (Figure 2B). Similar results were observed for the association between urinary arsenic and blood arsenic twelve weeks after baseline (Supplementary Figure 2, A and B; http://links.lww.com/EE/A205).

**Figure 3.**
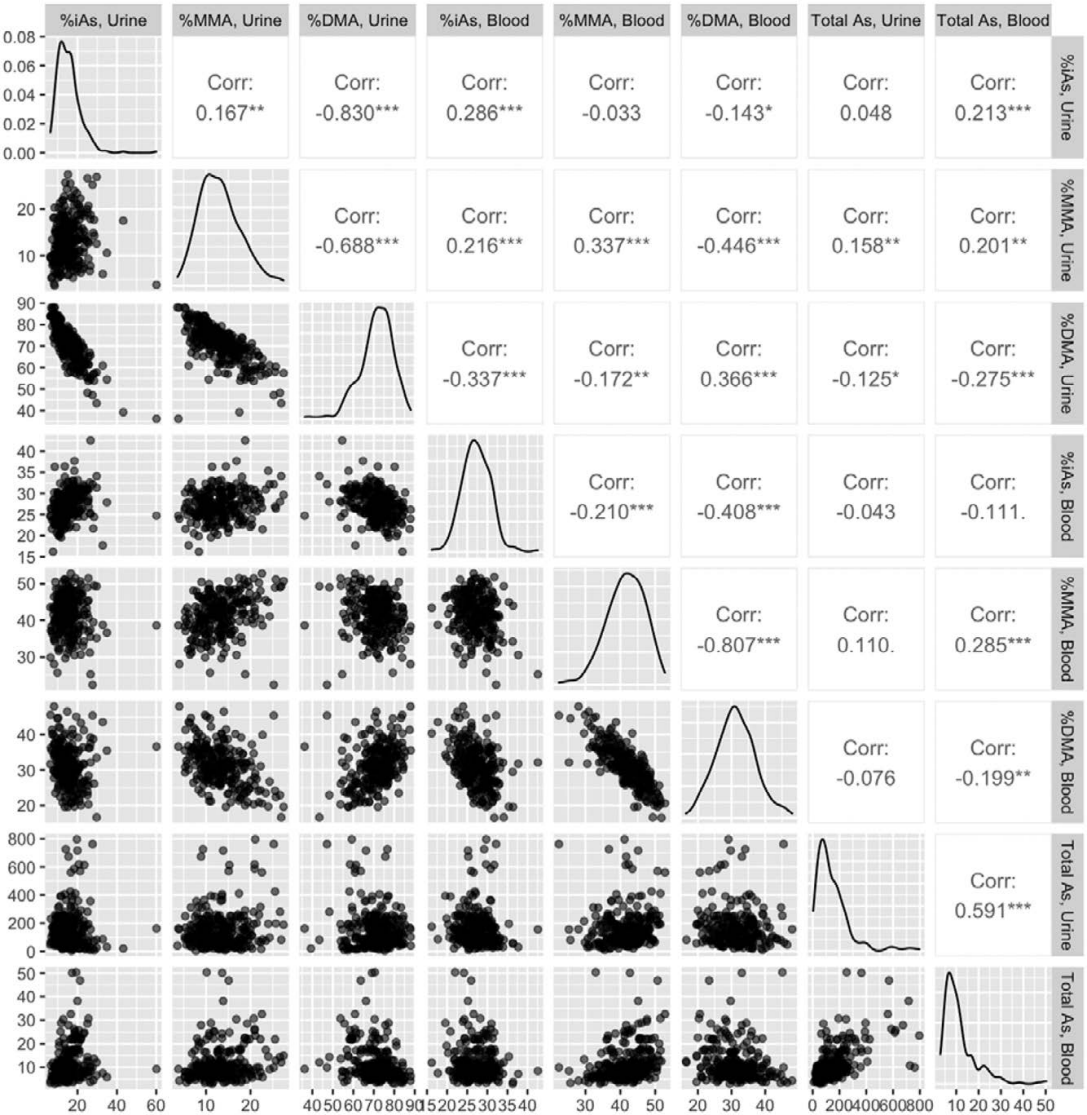
Scatterplot matrix of relative arsenic species abundances and total arsenic in blood and urine, with corresponding Pearson correlation coefficients.

The proportion of each arsenic species (DMA%, MMA%, and iAs%) detected in blood was positively associated with the proportion of the same species detected in urine (*P* = 1.5 × 10^–9^, *P* = 2.9 × 10^–7^, *P* = 1.3 × 10^–6^ respectively), with a positive correlation observed for each arsenic species (DMA%: r = 0.37, MMA%: r = 0.34, iAs%: r = 0.29) (Figure 3). Adjusting for sex, BMI, and smoking status did not appreciably change the association results (not shown).

In urine, a higher concentration of total arsenic was associated with a lower relative abundance of DMA (*P* < 1.2 × 10^–7^). A similar association was observed for total arsenic and relative abundance of DMA measured in blood (*P* < 5.6 × 10^–4^). Changes in model estimates after adjusting for sex, BMI, and smoking status were minimal.

### Associations with demographic variables

The proportion of each arsenic species assessed at baseline (week 0) and at week 12 were both used (in a mixed-effects model) to assess the association between relative metabolite abundance and demographic and lifestyle variables (Table 2). Male sex was associated with lower DMA% (in both urine and blood), higher MMA% (urine and blood), and lower total arsenic (urine). Age was associated with higher MMA% (urine). BMI was associated with higher DMA% (urine), lower MMA% (urine), and iAs% (urine). The associations observed were generally consistent across the multiple timepoints for each demographic variable (Supplementary Table 1; http://links.lww.com/EE/A205).

**Table 2.** Associations of demographic and lifestyle variables with relative abundance of arsenic species measured in both blood and urine

	**Regression coefficient, β (*P* value**)^a^			
Arsenic Species^b^	Sex (male)	Age (years)	BMI (kg/m^2^)	Smoking Status (ever/never)
**DMA%**				
Urine	–2.9 (2.3 × 10^–3^)	–0.01 (0.80)	0.45 (1.1 × 10^–4^)	–0.17 (0.86)
Blood	–0.33 (0.63)	0.04 (0.21)	0.14 (0.17)	–1.59 (0.031)
**MMA%**				
Urine	2.9 (3.0 × 10^–9^)	0.06 (2.1 × 10^–3^)	–0.20 (8.5 × 10^–4^)	0.33 (0.51)
Blood	0.33 (0.60)	0.03 (0.30)	–0.12 (0.18)	1.27 (0.058)
**iAs%**				
Urine	–0.07 (0.92)	–0.05 (0.081)	–0.25 (5.5 × 10^–3^)	–0.16 (0.84)
Blood	–2.4 × 10^–4^ (1.0)	–0.07 (3.9 × 10^–4^)	–0.01 (0.82)	0.31 (0.50)
**Total As**				
Urine^c^	–11.7 (0.42)	–1.4 (0.02)	–5.4 (2.5 × 10^–3^)	42.9 (5.6 × 10^–3^)
Blood	0.35 (0.77)	–0.01 (0.89)	–0.14 (0.38)	3.0 (0.015)

^a^Multivariate model adjusted for age, sex, BMI, ancillary study, and smoking status

^b^Estimates are from mixed-effects models, as arsenic species were measured at week 0 and week 12, and both timepoints were analyzed together in a single model. Sample sizes for week 0: urinary total arsenic (n = 323), urinary arsenic species (n = 320), blood total arsenic and arsenic species (n = 275). Sample sizes for week 12: urinary total arsenic (n = 248), urinary arsenic species (n = 236), blood total arsenic and arsenic species (n = 204). Total observations: urinary total arsenic (n = 571), urinary arsenic species (n = 556), blood total arsenic and arsenic species (n = 479).

^c^Total urinary arsenic concentration (sum of iAs, MMA, and DMA) is creatinine-adjusted.

### Genetic associations with AME

Genetic association analyses were conducted for the proportion of each metabolite in both blood and urine in ~300 participants at baseline (week 0) and ~200 participants in week 12 (decrease in sample size due to the lack of follow-up in FOX, a cross-sectional study). Data from both week 0 and week 12 were used (in the context of a mixed model) to increase the statistical power of this association analysis. The results of these analyses are shown in Table 3. The direction of association between each SNP and the relative abundance of each metabolite was the same across both biospecimens and timepoints (Supplementary Table 2; http://links.lww.com/EE/A205). Nearly all of these associations had a *P* value <0.05 in the analyses leveraging both timepoints. We observed no clear associations between the four AME SNPs and creatinine-adjusted total urinary arsenic and total blood arsenic. We also failed to observe any association between each of the four AME SNPs and the absolute concentrations of each species (as opposed to the proportion of each species) in both blood and urine. When testing for interactions between sex and each SNP, we found no evidence of interaction; however, we have limited power to robustly identify SNP-sex interactions given the size of the dataset used for these analyses (Supplementary Table 3; http://links.lww.com/EE/A205).

**Table 3.** Associations between previously identified AME SNPs and relative concentrations of arsenic species measured in both urine and blood^c^

	**Regression coefficient, β (*P* value**)^a^			
Arsenic species^b^	rs61735836 (21:47572887)MAF(T): 6.5%	rs4919690 (10:104616500)MAF(C): 9.6%	rs11191492 (10:104747534)MAF(G): 16.1%	rs191177668 (10:104635687) MAF(T): 0.7%
**DMA%**				
** **Urine	–3.9 (1.2 × 10^–4^)	–2.6 (1.3 × 10^–3^)	2.1 (1.2 × 10^–3^)	–10.8 (1.3 × 10^–4^)
** **Blood	–2.7 (5.1 × 10^–4^)	–2.2 (2.0 × 10^–4^)	1.1 (0.03)	–4.4 (0.024)
**MMA%**				
** **Urine	1.0 (0.060)	1.4 (1.1 × 10^–3^)	–0.84 (0.01)	5.3 (2.6 × 10^–4^)
** **Blood	2.2 (1.6 × 10^–3^)	1.4 (0.01)	–0.90 (0.05)	2.9 (0.11)
**iAs%**				
** **Urine	2.9 (2.0 × 10^–4^)	1.2 (0.05)	–1.3 (0.01)	5.4 (0.01)
** **Blood	0.45 (0.36)	0.84 (0.03)	–0.19 (0.56)	1.55 (0.21)
**Total**				
** **Urine^a^	–6.1 (0.70)	–13.4 (0.29)	5.7 (0.59)	–18.3 (0.68)
** **Blood	–0.12 (0.92)	–0.89 (0.39)	–0.78 (0.36)	1.9 (0.59)

^a^Total urinary arsenic concentration (sum of iAs, MMA, and DMA) is creatinine adjusted.

^b^Estimates are from mixed-effects models, as arsenic species were measured at week 0 and week 12, and both timepoints were analyzed together in a single model. Sample sizes for week 0: urinary total arsenic (n = 301), urinary arsenic species (n = 290), blood total arsenic and arsenic species (n = 253). Sample sizes for week 12: urinary total arsenic and arsenic species (n = 222), blood total arsenic and arsenic species (n = 190). Total observations: urinary total arsenic (n = 523), urinary arsenic species (n = 444), blood total arsenic and arsenic species (n = 443).

^c^Multivariate model adjusted for age, sex, and ancillary study.

## Discussion

The primary objective of this study was to determine if SNPs known to associate with arsenic species composition in urine had similar effects on arsenic species measured in blood. Using data from arsenic-exposed individuals in Bangladesh, we show that all four genetic variants known to affect arsenic species in urine (3 near *AS3MT* and 1 in *FTCD*) also affect arsenic species composition in blood, with consistent directions of effect in both tissue types. Therefore, our results support the hypothesis that previously identified AME SNPs impact the efficiency of arsenic metabolism and elimination, and the internal dose of arsenic. The effect sizes observed for the *AS3MT* SNPs in urine are also comparable to those previously identified.^11^ The genetic effects on arsenic species in blood suggest that these variants may impact exposure burden across multiple toxicity-prone organs, as blood interacts with all human organs and tissues.

Few prior studies have attempted to examine the impact of inherited AME-related variants on arsenic species measured in blood. One prior paper in HEALS attempted such analyses (Pierce et al. 2019), but the sample size was <50% of that reported here and was, therefore, underpowered to detect any clear associations for SNPs in the *AS3MT* region. In this study, we are able to confirm that genetic effects on the relative distribution of arsenic species present in urine are similarly observed in blood, a human tissue type that interacts with many other tissues types and organs, including those prone to arsenic toxicity.

Secondary objectives of this study included the comparison of arsenic species composition in blood and urine, as well as assessing their association with participant characteristics. We observed clear correlation between relative arsenic species concentrations measured in blood samples and those measured in urine samples (0.20 < r < 0.39), as reported previously.^20,32^ However, there are several key differences between urine and blood. First, the proportion of DMA in urine samples (~72% on average) was much larger than the proportion observed in blood samples (~31%),^8^ likely due to the known shorter circulating half-life of DMA. Additionally, the association of demographic and lifestyle factors with arsenic species variables was generally consistent for blood and urine, particularly for sex. We did not observe clear patterns of association for age and smoking status, likely due to limited sample size and power. We also observed greater variability in urinary arsenic metabolites compared with blood arsenic metabolites, as more outliers were observed based on urine measurements (Figure 1B). Other research has established that blood As metabolite concentrations tend to be more tightly regulated than urine metabolite concentrations and therefore urine may provide information about metabolic capacities that are not detected in blood.^33,34^

A previous study of HEALS participants conducted by Jansen et al. estimated the association between demographic characteristics and arsenic species in urine (rather than blood) using a larger dataset of 4,073 participants.^35^ Our work uses a subset of these participants, but it relies on an independent set of biospecimens and arsenic measurements (taken as a part of the FOX, FACT, and NIAT studies, rather than the HEALS parent study). The associations we observed for sex and BMI were consistent with those reported by Jansen et al. However, we did not observe clear associations of arsenic species composition with age or smoking status. It is possible that our analysis was underpowered to detect the association with age reported by Jansen, et al., as the sample size of that study was >4,000. Furthermore, our results related to urine arsenic species are generally consistent with those reported previously, including the associations observed for SNPs rs4919690 (previously represented by rs9527) and rs11191492 (previously represented by rs11191527). Among the SNPs studied in this work, those with lower minor allele frequencies (e.g., FTCD rs61735836 MAF = 6.5%, AS3MT rs4919690 MAF = 0.7%) tended to have larger effect sizes. Since statistical power in GWA studies decreases as minor allele frequency decreases, this relationship may be explained by GWA studies’ low power to detect rare variants with weak effects. Similar relationships have also been observed in past GWA studies.^36–38^

Our study is limited by sample size, with only 334 participants included limiting the statistical power of our analyses. However, the primary goal of this study was to assess the consistency of genetic associations previously reported for arsenic species measured in urine (rather than identification of SNPs based on a genome-wide search). Thus, because the direction and magnitude of associations with arsenic species in blood appears comparable to those observed for arsenic species in urine, the small sample size of our sample was not detrimental to our analysis for this study. Our use of multiple timepoints for each individual mitigated potential issues with sample size and power.

Our data from FACT and NIAT also includes arsenic measurements taken after folate-related interventions to boost AME. Given our study’s small sample size and the large number of intervention arms (eight in total) in FACT and NIAT, it was not feasible to examine SNP-treatment interactions. However, it is highly unlikely that our results are confounded by treatment group, as treatment assignment is randomized in each study. Furthermore, estimates at baseline (Week 0) are relatively consistent with those observed at Week 12, implying that the SNP effects observed were not substantially modified by the intervention.

In this study, we did not specifically examine dietary sources of inorganic or organic arsenic exposure, which could potentially contribute to DMA.^14–16,26^ However, HEALS baseline urinary total arsenic is very strongly correlated with baseline measurements of inorganic arsenic in participants’ primary wells used for drinking water (r = 0.76).^39^ This observation suggests that the majority of arsenic exposure (and DMA) present in HEALS participants’ urine samples is from water sources. Diet is likely to be a larger contributor to arsenic exposure in populations with low exposure through drinking water. In HEALS, the high levels of inorganic arsenic in drinking water likely minimize the contribution of diet to total arsenic exposure.^40^

In conclusion, we found that SNPs which influence arsenic species composition in urine (i.e., AME) have similar effects on arsenic species measured in blood. More specifically, for all four SNPs examined, the allele showing a negative association with DMA% in urine also showed a negative association with DMA% in blood. These DMA%-decreasing alleles have been shown previously to be associated with increased risk of arsenic-related skin lesions in HEALS.^12^ The consistency we observe provides further support for the hypothesis that AME SNPs identified to date impact the efficiency of arsenic metabolism and elimination, thereby affecting internal dose of arsenic (and circulating metabolite levels) and potentially impacting exposure across multiple toxicity-prone organs.

Although this work does not identify novel variants (for risk assessment purposes), it provides additional evidence that arsenic metabolism efficiency SNPs impact internal exposure across multiple tissue types. These results provide us with increased confidence that the genetic information we currently collect for risk assessment is relevant to arsenic toxicity risk across multiple toxicity-prone organs and tissue types, especially because blood interacts with all organs and tissue types. Recent work has also demonstrated that AME SNPs near AS3MT regulate AS3MT expression in most human tissue types,^41^ further suggesting an important role for AME-related SNPs in diverse tissue types.

This work was conducted on a Bangladeshi population with high exposure to arsenic; therefore, the extent to which our findings are generalizable to other populations remains unclear. Future studies should examine other populations with different levels of exposure (and different ancestry) to assess the generalizability of our findings. Larger studies of genetic effects on arsenic species in blood and other tissue types are needed to further validate our findings across tissues, explore the possibility of tissue-specific genetic effects on arsenic metabolism, and investigate any influence SNPs may have on the efficacy of arsenic metabolism interventions.

## Acknowledgments

We would like to thank all the study participants and staff who have contributed to HEALS, NIAT, FOX, and FACT.

## Supplementary Material


