# Supplementary material for: Inherited genetic effects on arsenic metabolism: A comparison of effects on arsenic species measured in urine and in blood
Source: Environ Epidemiol. 2022 Nov 11;6(6):e230. doi: 10.1097/EE9.0000000000000230 (PMC9746746; doi:10.1097/EE9.0000000000000230)
Supplement: Supplementary file 1 [file ee9-6-e230-s001.docx]

**Supplementary Materials**

| A  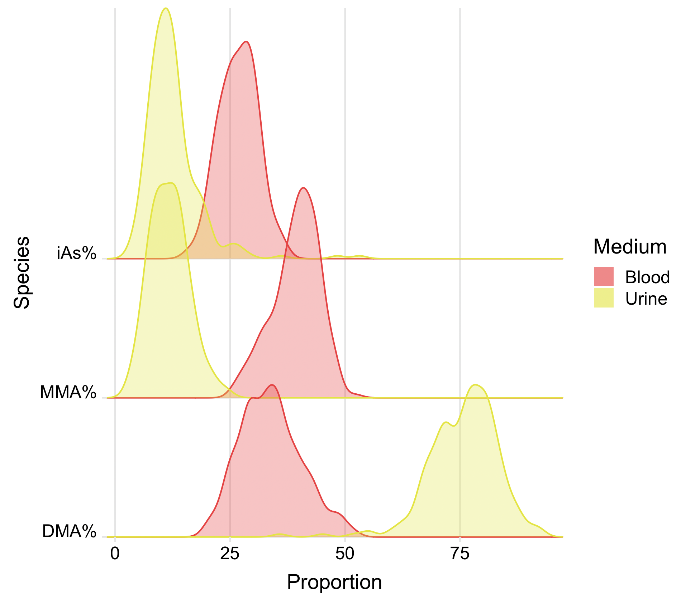 | 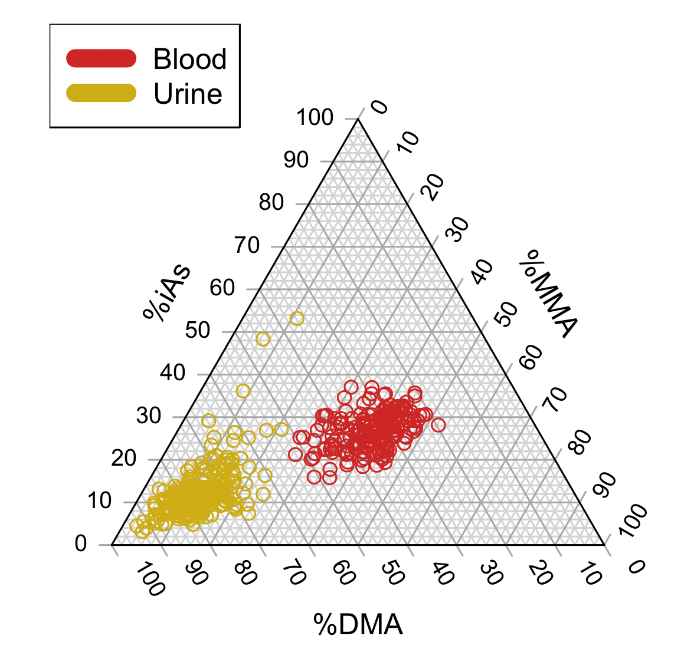B |
| --- | --- |
| **Supplementary Figure 1**. Distribution of arsenic species percentages in blood (red) and urine (yellow/gold) for arsenic measurements twelve weeks after baseline measurements. Ridge plot (A) Triangle plot (B) | |

| A  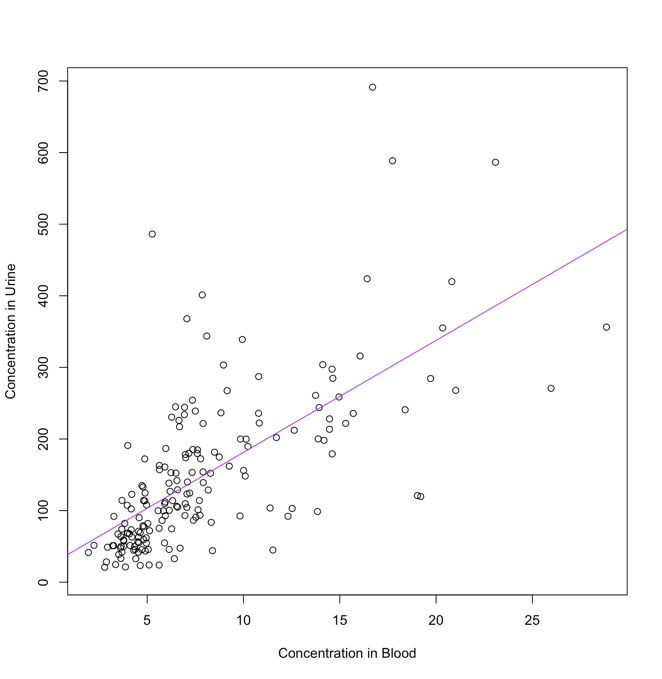 | B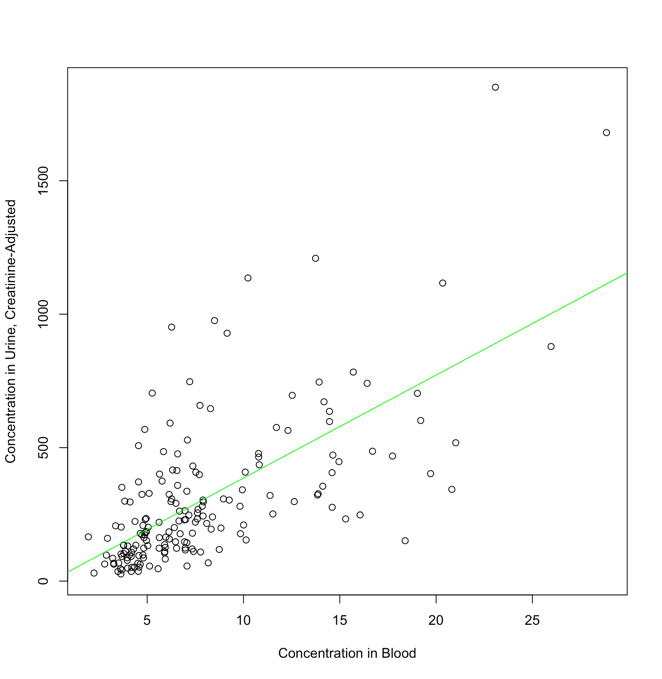 |
| --- | --- |
| **Supplementary Figure 2**. Association between urinary arsenic and blood arsenic before (A) and after creatinine adjustment (B) twelve weeks after baseline measurements. Blood and urine total arsenic was estimated by taking the sum of iAs, MMA, and DMA. | |

| **Supplementary Table 1.** Associations of demographic and lifestyle variables with relative abundances of arsenic species measured in both urine and blood | | | | |  |
| --- | --- | --- | --- | --- | --- |
|  | **Regression coefficient, β (p-value)***** | | | |  |
| **Arsenic Species** | **Sex (male)** | **Age (years)** | **BMI (kg/m^2^)** | **Smoking Status (ever/never)** | |
| **Week 0***  **DMA%** |  |  |  |  | |
| urine | -3.0 (0.018) | -0.009 (0.85) | 0.51 (1.0 x 10^-3^) | -0.41 (0.75) | |
| blood | -0.65 (0.45) | 0.022 (0.54) | 0.15 (0.21) | -1.99 (0.03) | |
| **MMA%** |  |  |  |  | |
| urine | 2.6 (9.9 x 10^-5^) | 0.069 (0.011) | -0.23 (6.3 x 10^-3^) | 0.67 (0.34) | |
| blood | 0.68 (0.39) | 0.023 (0.51) | -0.11 (0.30) | 1.85 (0.03) | |
| **iAs%** |  |  |  |  | |
| urine | -0.26 (0.79) | -0.060 (0.12) | -0.28 (0.015) | -0.26 (0.79) | |
| blood | -0.028 (0.96) | -0.045 (0.061) | -0.037 (0.63) | 0.14 (0.82) | |
| **Total** |  |  |  |  | |
| urine** | -19.4 (0.34) | -1.6 (0.049) | -5.8 (0.017) | 42.9 (0.044) | |
| blood | 0.35 (0.77) | -0.007 (0.89) | -0.14 (0.38) | 3.04 (0.015) | |
| **Week 12*** |  |  |  |  | |
| **DMA%** |  |  |  |  | |
| urine | -2.8 (0.043) | -0.012 (0.84) | 0.38 (0.027) | -0.12 (0.93) | |
| blood | 0.09 (0.93) | 0.054 (0.24) | 0.11 (0.49) | -1.04 (0.36) | |
| **MMA%** |  |  |  |  | |
| urine | 3.3 (2.4 x 10^-6^) | 0.05 (0.068) | -0.17 (0.052) | -0.10 (0.89) | |
| blood | -0.12 (0.90) | 0.038 (0.36) | -0.12 (0.36) | 0.47 (0.64) | |
| i**As%** |  |  |  |  | |
| urine | -0.59 (0.60) | -0.040 (0.37) | -0.21 (0.13) | -0.027 (0.98) | |
| blood | 0.029 (0.97) | -0.092 (2.2 x 10^-3^) | 0.021 (0.83) | 0.57 (0.43) | |
| **Total** |  |  |  |  | |
| urine** | -1.4 (0.95) | -1.06 (0.25) | -4.9 (0.066) | 43.4 (0.054) | |
| blood | 0.62 (0.50) | -6.6 x 10^-3^ (0.87) | -0.40 (3.9 x 10^-3^) | 1.14 (0.25) | |
| * Estimates are from multivariate linear regression models, as arsenic species were measured at week 0 and week 12, and both timepoints were analyzed in separate models. Sample Sizes: week 0 (total urine: n = 323, urinary metabolites: n = 320, total blood and blood metabolites: n = 275), week 12 (total urine: n = 248, urine: n = 236, total blood and blood metabolites: n = 204)  **Total urinary arsenic concentration (sum of iAs, MMA and DMA) is creatinine-adjusted.  ***Multivariate model adjusted for age, sex, BMI, ancillary study, and smoking status | | | | | |

| **Supplementary Table 2.** Associations between previously-identified AME SNPs with relative abundances of arsenic species measured in both urine and blood | | | | |  |
| --- | --- | --- | --- | --- | --- |
|  | **Regression coefficient, β (p-value)***** | | | |  |
| **Arsenic Species** | **rs61735836**  **(21:47572887)**  **MAF (T): 6.5%** | **rs4919690**  **(10:104616500)**  **MAF (C): 9.6%** | **rs11191492**  **(10:104747534)**  **MAF (G): 16.1%** | **rs191177668**  **(10:104635687)**  **MAF (T): 0.66%** | |
| **Week 0***  **DMA%** |  |  |  |  | |
| urine | -5.0 (2.1 x 10^-4^) | -3.6 (6.9 x 10^-4^) | 1.9 (0.034) | -14.2 (3.7 x 10^-4^) | |
| blood | -2.7 (4.9 x 10^-3^) | -2.2 (3.7 x 10^-3^) | 1.2 (0.051) | -3.4 (0.19) | |
| **MMA%** |  |  |  |  | |
| urine | 1.4 (0.055) | 1.6 (4.2 x 10^-3^) | -0.83 (0.084) | 7.2 (7.3 x 10^-4^) | |
| blood | 1.5 (0.077) | 1.6 (0.021) | -1.1 (0.048) | 2.3 (0.35) | |
| **iAs%** |  |  |  |  | |
| urine | 3.6 (3.5 x 10^-4^) | 2.0 (0.013) | -1.1 (0.11) | 7.0 (0.020) | |
| blood | 1.1 (0.063) | 0.59 (0.22) | -0.09 (0.83) | 1.2 (0.48) | |
| **Total** |  |  |  |  | |
| urine** | -25.5 (0.25) | -10.8 (0.53) | -8.4 (0.56) | 14.4 (0.82) | |
| blood | -0.12 (0.93) | -0.9 (0.39) | -0.78 (0.36) | 1.9 (0.59) | |
| **Week 12*** |  |  |  |  | |
| **DMA%** |  |  |  |  | |
| urine** | -2.4 (0.10) | -1.2 (0.32) | 2.4 (9.4 x 10^-3^) | -7.3 (0.051) | |
| blood | -2.6 (0.024) | -2.3 (0.01) | 0.87 (0.25) | -5.6 (0.042) | |
| **MMA%** |  |  |  |  | |
| urine | 0.44 (0.56) | 0.96 (0.11) | -0.85 (0.077) | 3.4 (0.071) | |
| blood | 3.1 (2.9 x 10^-3^) | 1.1 (0.17) | -0.56 (0.41) | 3.6 (0.14) | |
| i**As%** |  |  |  |  | |
| urine | 2.0 (0.098) | 0.21 (0.82) | -1.6 (0.038) | 3.9 (0.20) | |
| blood | -0.47 (0.54) | 1.16 (0.047) | -0.31 (0.52) | 2.0 (0.27) | |
| **Total** |  |  |  |  | |
| urine** | 20.5 (0.38) | -16.8 (0.36) | 23.4 (0.12) | -52.4 (0.38) | |
| blood | 2.2 (0.036) | -1.0 (0.20) | -0.57 (0.40) | -0.85 (0.73) | |
| * Estimates are from multivariate linear regression models, as arsenic species were measured at week 0 and week 12, and both timepoints were analyzed in separate models. Sample Sizes: week 0 (total urine: n = 323, urinary metabolites: n = 320, total blood and blood metabolites: n = 275), week 12 (total urine: n = 248, urine: n = 236, total blood and blood metabolites: n = 204)  **Total urinary arsenic concentration (sum of iAs, MMA and DMA) is creatinine-adjusted.  *** Multivariate model adjusted for age, sex, and ancillary study | | | | | |

| Supplementary Table 3: Interaction between previously-identified AME SNPs and sex measured in both urine and blood** | | | | | | |  |
| --- | --- | --- | --- | --- | --- | --- | --- |
|  | **Regression coefficient, β (p-value)***** | | | | | |  |
| **Arsenic Species*** | **rs61735836** **(21:47572887)**  **MAF (T): 6.5%** | **rs61735836xSex (21:47572887xSex)**  **MAF (T): 6.5%** | **rs4919690 (10:104616500) MAF (C): 9.6%** | **rs4919690xSex (10:104616500xSex)**  **MAF (C): 9.6%** | **rs11191492 (10:104747534)**  **MAF (G): 16.1%** | **rs11191492xSex**  **(10:104747534xSex)**  **MAF (G): 16.1%** | |
| **DMA%** |  |  |  |  |  |  | |
| urine | -5.19 (4.60 x 10^-4^) | 2.49 (0.22) | -0.75 (0.59) | -2.69 (0.11) | 1.96 (0.035) | 0.37 (0.778) | |
| blood | -2.32 (0.040) | -0.61 (0.69) | -0.74 (0.48) | -2.1 (0.087) | 0.91 (0.181) | 0.40 (0.683) | |
| **MMA** |  |  |  |  |  |  | |
| urine | 1.68 (0.032) | -1.26 (0.23) | -0.087 (0.90) | 2.13 (0.016) | -0.33 (0.49) | -1.03 (0.132) | |
| blood | 1.80 (0.080) | 0.72 (0.60) | 0.022 (0.98) | 1.99 (0.088) | -0.30 (0.62) | -1.29 (0.149) | |
| **iAs%** |  |  |  |  |  |  | |
| urine | 3.59 (1.97 x 10^-3^) | -1.23 (0.43) | 0.85 (0.44) | 0.55 (0.68) | -1.64 (0.023) | 0.66 (0.51) | |
| blood | 0.52 (0.48) | -0.12 (0.90) | 0.72 (0.28) | 0.18 (0.82) | -0.60 (0.16) | 0.89 (0.15) | |

* Estimates are from mixed effects models, as arsenic species were measured at week 0 and week 12, and both timepoints were analyzed together in a single model. Sample sizes for week 0: urinary arsenic species (n = 290), blood arsenic species (n = 253). Sample sizes for week 12: urinary arsenic species (n = 222), blood arsenic species (n = 190).

**rs191177668 (10:104635687) not included because the only subjects with this SNP were male, so it was not possible to test for an interaction

*** Multivariate model adjusted for age, sex, and ancillary study
